# Allopurinol-Induced Stevens–Johnson Syndrome in Javanese Men With Positive HLA‐B*58:01

**DOI:** 10.3389/fgene.2022.839154

**Published:** 2022-06-13

**Authors:** Astri Ferdiana, Jajah Fachiroh, Dyah Ayu Mira Oktarina, Astrid Irwanto, Caroline Mahendra, Sri Awalia Febriana, Hardyanto Soebono

**Affiliations:** ^1^ Department of Public Health Faculty of Medicine, Universitas Mataram, Mataram, Indonesia; ^2^ Center for Tropical Medicine, Faculty of Medicine Public Health and Nursing Universitas Gadjah Mada, Yogyakarta, Indonesia; ^3^ NLR Indonesia, Jakarta, Indonesia; ^4^ Department of Histology and Cell Biology, Faculty of Medicine Public Health and Nursing Universitas Gadjah Mada, Yogyakarta, Indonesia; ^5^ Department of Dermatology and Venereology, Faculty of Medicine Public Health and Nursing Universitas Gadjah Mada, Yogyakarta, Indonesia; ^6^ Nalagenetics Pte Ltd., Singapore, Singapore

**Keywords:** allopurinol, stevens-johnson syndrome, adverse drug reaction, HLA-B*58:01, pharmacogenetics, severe cutaneous adverse reactions

## Abstract

**Background:** Allopurinol is the most commonly used drug for the treatment of gout arthritis. However, the use of allopurinol is associated with severe cutaneous adverse reactions (SCARs) and life-threatening immune-mediated reactions that include Stevens–Johnson syndrome (SJS). SJS induced by allopurinol is strongly linked with the presence of HLA-B*58:01 in the Asian population. Such a study has not been conducted in Indonesia. We present two cases with clinical diagnosis of SJS. These patients had Javanese ethnicity, for which evidence on the genetic predisposition of allopurinol-induced SJS/TEN had not been established. Testing for the presence of the *HLA-B∗58:01* allele was positive in both cases. Our case report confirms findings from studies in Asian countries that link HLA-B*58:01 and allopurinol-induced SJS/TEN. A larger study is needed to elicit evidence that the HLA-B*58:01 allele can potentially be used as a genetic marker for allopurinol-induced SCARs among different ethnicities in Indonesia.

## Introduction

Allopurinol is the main therapeutic agent for the treatment of gout arthritis, a condition caused by an elevated blood urate level, and is becoming a major problem in many countries. Allopurinol inhibits xanthine oxidase, an enzyme involved in the oxidation of hypoxanthine and xanthine, reactions that ultimately result in the production of uric acid (Stamp et al., 2016). In 2017 alone, more than 14 million prescriptions of allopurinol were dispensed in the United States, making it the most widely prescribed medicine for gout arthritis (Russell et al., 2020).

However, the use of allopurinol is associated with adverse drug effects that can range from a mild form of hypersensitivity with maculopapular eruption (MPE) to severe cutaneous adverse reactions (SCARs), life-threatening immune-mediated reactions. SCARs induced by allopurinol include Stevens–Johnson syndrome (SJS), toxic epidermal necrolysis (TEN), drug reaction with eosinophilia and systemic symptoms (DRESS), and the systemic manifestations of allopurinol hypersensitivity syndrome (AHS) (Stamp et al., 2016; Sukasem et al., 2016). These reactions are mediated by delayed type IV hypersensitivity reactions due to T cell–mediated drug-specific immune response (Bellón, 2019).

Prior studies demonstrate the human leukocyte antigen (HLA) genetic predisposition to allopurinol-induced SCARs (Somkrua et al., 2011). A strong association between HLA-B*58:01 and allopurinol-associated SJS/TEN has been discovered in different ethnic groups including Asian (Somkrua et al., 2011), Caucasian (Chang et al., 2020), and African American (Fontana et al., 2021). The association between HLA-B*58:01 and allopurinol-induced SJS/TEN is, however, stronger in the Asian population, which is indicated by a positivity rate of 100% in the Asian population compared to 60% in Caucasian origins (Low et al., 2020).

In Indonesia, SCARs account for around one third of the overall adverse cutaneous drug reactions (ACDR) with a mortality rate of around 5% of all cases (Oktarina et al., 2015). A previous study suggests that the HLA-B*58:01 is a strong risk factor for allergy due to nevirapine among individuals with HIV/AIDS in Indonesia (Pudjiati et al., 2016). However, the relationship between HLA-B*58:01 and allopurinol-induced SJS/TEN in the Indonesian population has not been established despite the relatively high frequency of this allele among Javanese ethnics (Saito et al., 2016), which accounts for 40% of the Indonesian population (Portal Informasi Indonesia, 2022). In the current study, we describe the features and clinical outcomes in two patients of Javanese ethnicity with manifestations of allopurinol-induced SJS/TEN, which is demonstrated to be associated with the HLA-B*58:01 allele.

## Case Presentation

### Patient One

A 63-year-old man with a chief complaint of rash was admitted at the emergency room of the Sardjito General Hospital in Yogyakarta, Indonesia. His Javanese ethnicity was confirmed from parents and both grandparents of the patient. Upon physical examination, the temperature was 37°C, arterial blood pressure was 130/80 mm Hg, and heart rate was 87 beats/min. Further physical examination revealed diffuse maculopapular erythema on almost the whole body (more than 30%), some erosion, and denuded skin on the chest and lower extremities. Erosion was also found on the mucosal areas, such as lip, conjunctiva, and genital area, including scrotum and external urethral orifice with pain at urination. As the findings of clinical examination, i.e., skin and mucosal involvement and positive Nicolsky’s sign, were consistent with SJS, diagnosis of SJS was established. Upon further history taking, the patient had a history of gout arthritis and allopurinol use. He had taken 200 mg/day allopurinol 2 months before skin lesion erupted. He also took metamizole and diazepam 2 months before the occurrence of symptoms.

Laboratory examination revealed normal blood counts and hepatic enzymes but markedly elevated ureum and creatinine ratio, suggesting decreased renal function (Table 1). All medications were discontinued, and the patient was administered methylprednisolone of 125 mg/24 h for 4 days. After 2 days, the dosage was tapered off to 62 mg/24 h for 2 days. The patient was also treated with ibuprofen, cetirizine, tramadol, and topical preparation. Dressing with NaCl 0.9% was applied for 15 min on a daily basis. No new erythematous patches or vesiculobullous lesions were observed upon discharge.

**TABLE 1:** Laboratory examination results at admission.

Parameters	Patient 1 (63 years old, male)	Patient 2 (43 years old, male)	Normal range
Haemoglobin	14.1	9	13–17 g/dl
Erythrocyte count	4.91	3,09*	4.7–6.1 cells/L
Leukocyte count	9.68*	11,8*	3.5–9.5 × 10^9^ cells/L
Neutrophile	86.6*	80,4*	40–75%
Monocyte	5.3	9,8	3–10%
Lymphocyte	8	5,5	20–50%
Basophile	0.1	0,1	0–1%
Eosinophile	0.5	1,2	0.4–8%
Albumin	2.7	3.56	3.5–5.5 g/dl
Aspartate aminotransferase	33	83*	15–40 U/L
Alanine aminotransferase	27	33	9–50 U/L
Blood urea nitrogen (BUN)	64.6*	87,9*	7–20 mg/dl
Creatinine	1.64*	7.52*	0.6–1.2 mg/dl
Blood glucose	132	112	80–120 mg/dl

### Patient Two

A 43-year-old man was admitted at the emergency room of the Sardjito General Hospital with a chief complaint of rash on the overall body. The ethnicity was Javanese, based on the ethnicity of parents and both grandparents. Vital sign examination showed an elevated blood pressure of 170/100 mmHg, heart rate of 80 beats/min, respiratory rate of 20/min and normal temperature.

**FIGURE 1:**
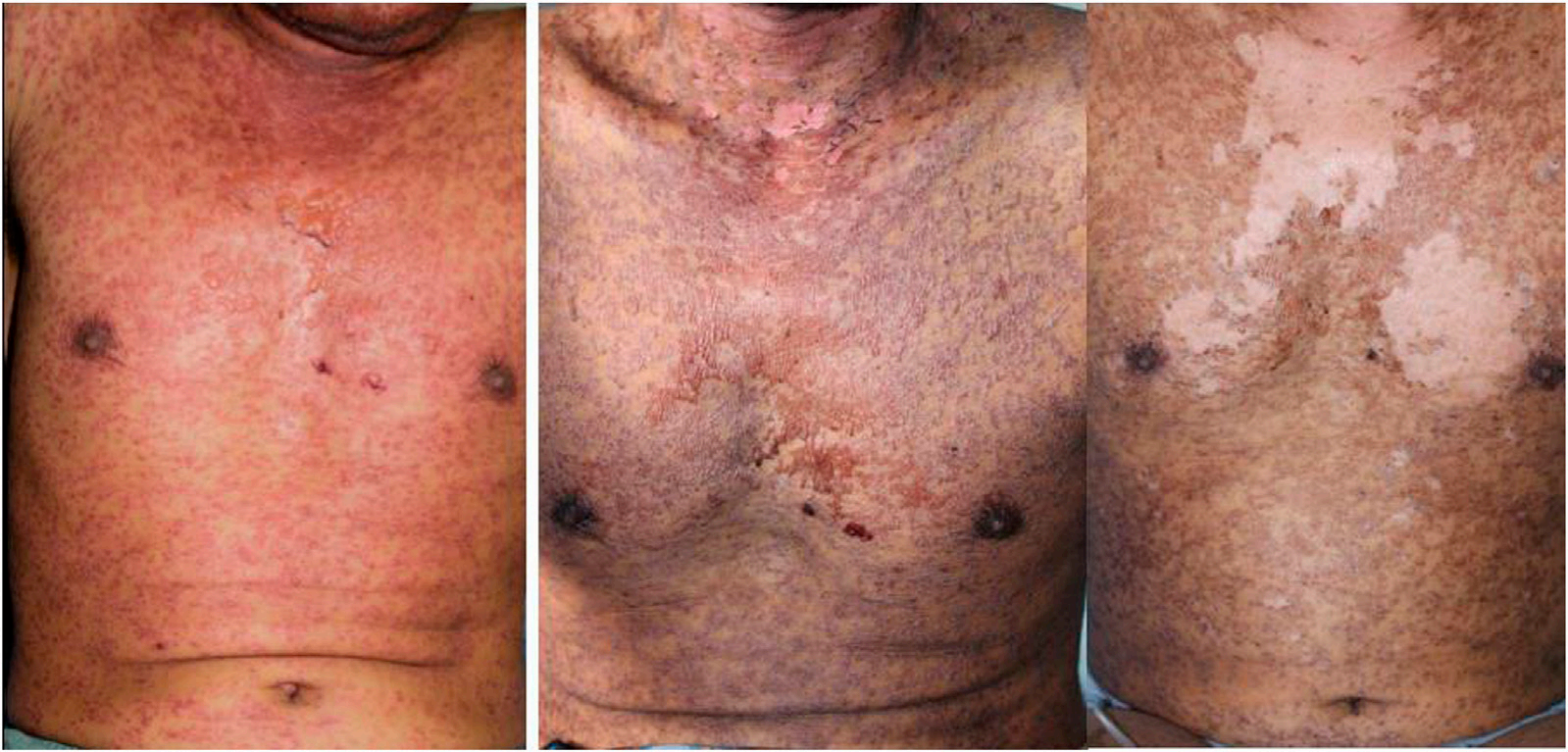
Maculopapular erythema with erosions, leaving a denuded skin on the chest.

Three days prior to the admission, he complained of fever, headache, muscle pain, and took over-the-counter medicine. Two days before the admission, rashes occurred on the face in addition to red eyes and mouth ulcers. These symptoms got worse on the day before the admission when the rash spread to the overall body. Physical examination showed erythema on the overall body (more than 30%), eye discharge, and mouth ulcers (Figure 1). Laboratory examination showed an increased leukocyte count with increased percentage of neutrophil, decreased erythrocyte counts, and markedly elevated liver enzymes and renal function tests (Table 1).

The diagnosis of SJS was established based on the skin and mucosal involvement and positive Nicolsky’s sign. One month before the admission, the patient had started allopurinol and antihypertensive medication. The patient had a history of hypertension in the last year before the complaints started. The patient was administered methylprednisolone of 31.25 mg/24 h. Allopurinol was discontinued, but antihypertensive medications were continued. Dressing with NaCl 0.9% was given for 15 min twice a day. At discharge, the patient did not show either new erythematous patches or vesiculobullous lesions.

## Genetic Testing for HLA

Three ml peripheral blood was sampled from both patients by using an EDTA-containing vacutainer from each patient. Blood samples were transported to the Molecular Biology Laboratory of the Faculty of Medicine, Public Health and Nursing Universitas Gadjah Mada. Buffycoat was isolated from blood samples by 1500 RPM cold centrifugation for 10 min. Further, DNA was extracted from 100 ul of buffycoat by using a QIAamp^(R)^ DNA minikit (Qiagen, Switzerland) by following the instruction manual. The quality of extracted DNA was analyzed through DNA electrophoresis with the objective to observe single genomic DNA as well as 260/280 nm absorbance ratio of ≥1.75 by using a spectrophotometer (DeNovix DS-11). The quantity of DNA was obtained through multiplication of 50 ug/ml × 260 nm spectrophotometer read-out × dilution factor. Further, purified DNA was stored in a −20°C freezer until used. Purified DNA was transported in cold chain to the School of Medicine and Health Sciences, Atma Jaya Catholic University of Indonesia, for further downstream genomic analysis.

HLA-B*58:01 was determined by using the ExProbe™ HLA B*58:01 Typing Kit (TBG Biotechnology Corp., Taipei, Taiwan) following its instruction manual. The detection system was based on the use of a real-time quantitative polymerase chain reaction (qPCR) technique containing primer mixes and SYBR green dyes. The presence of amplification is detected by activation of fluorescence and a positive indication of the existence of allele specific sequence within the genomic DNA. An internal control primer pair that amplifies a conserved region of the housekeeping gene, cystic fibrosis gene, was included in the PCR reaction mix, and the PCR product of the internal control primer pair serves as an indication of the integrity of the PCR reaction. A negative control was also included in the kit, which should provide a negative result after the completion of the PCR.

For each PCR reaction, a mix of 4.5 ul primer mix, 10.5 ul qPCR buffer mix, and 3 ul of template DNA was made, containing either positive or negative controls or purified DNA at concentrations ranging from 5 to 40 ng/μl. The thermal cycling was run using the BioRad CFX96 Real Time Detection System programmed as follows: one cycle of heating at 95°C for 3 min, 36 cycles of denaturation at 93°C for 30 s, annealing at 62°C for 45 s, and elongation at 72°C for 40 s. An additional step done for data collection included recording Melt Curve between 65°C and 95°C for 15 s, at increments of 0.5°C. Upon run completion, the melt peak threshold was set at 40 -dRFU/dT to determine melt temperature when peaks were present.

**FIGURE 2:**
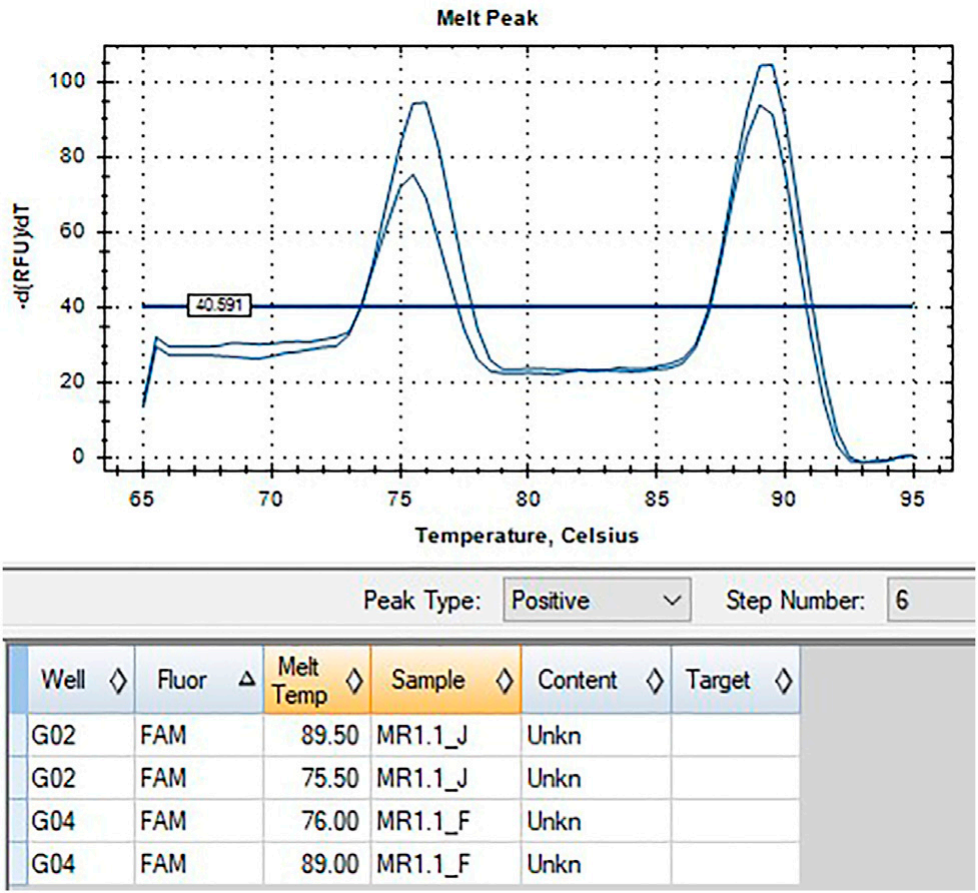
qPCR melt curve analysis and quantification amplification result of Patient 1.

**FIGURE 3:**
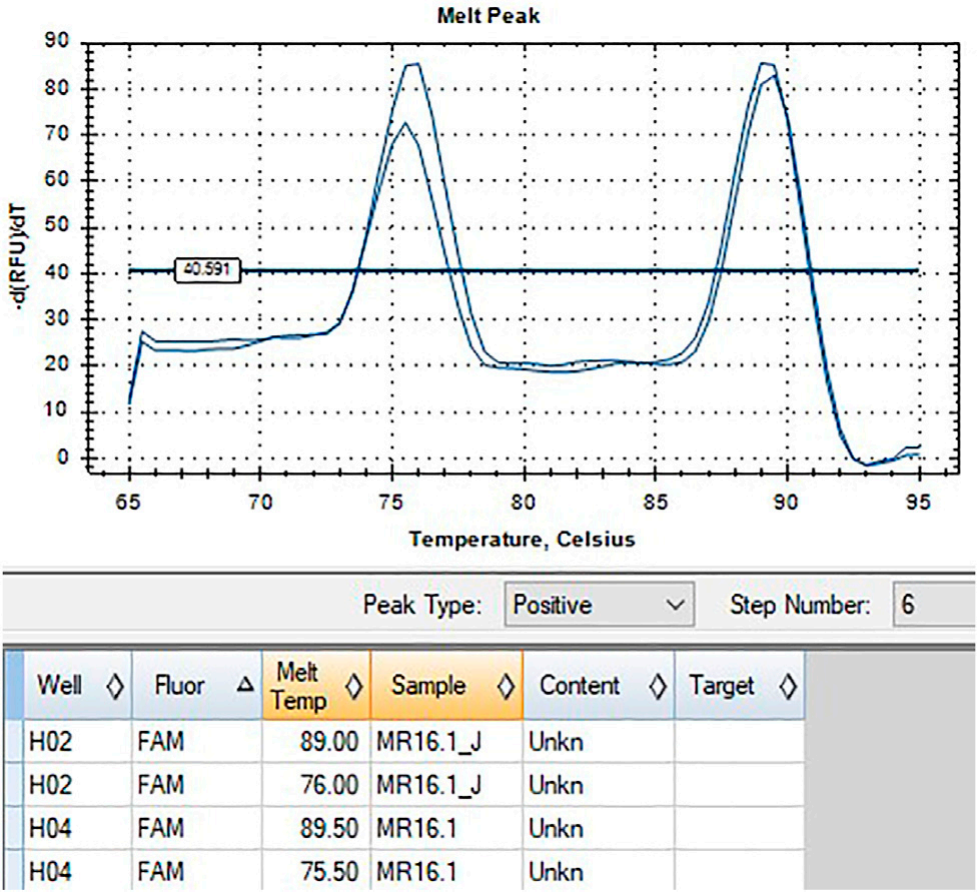
qPCR melt curve analysis and quantification amplification result of Patient 2.

Indication of the HLA-B*58:01 allele was observed as the presence of peaks in the melt curve plot profile of the internal control and target allele region at 73.5°C–79°C and 88.5°C–90°C, respectively, or a single peak at 88.5°C–90°C. Whereas the absence of HLA-B*58:01 is shown by a presence of peak at 73.5°C–79°C denoting internal control only, but not the target allele region. HLA-B*58:01 typing of both samples showed positive results (Figures 2, 3).

### Ethical Consideration

Ethical approval was obtained from the Medical Research Ethics Committee at Universitas Gadjah Mada no KE/FK/1150/EC. Written consent was obtained from each patient after receiving adequate information about the study.

## Discussion

We presented two cases of SJS and SJS//TEN associated with a history of allopurinol use. Allopurinol-induced SCARs typically occur in the first few weeks or months after starting allopurinol. The median time of onset of this severe reaction was 3 weeks (Ka et al., 2019). In our cases, patients had been taking allopurinol for their gout arthritis condition for 1–2 months prior to the onset of skin eruption.

In Indonesia, allopurinol as a xanthine inhibitor is the first line of drugs in the management of gout arthritis to lower uric acid levels (Perhimpunan Reumatologi Indonesia, 2018). Gout arthritis is the most common inflammatory arthritis caused by depositions of urine crystals on the joints of feet, knees, elbows, and hands. Regular treatment with allopurinol is beneficial as a long-term management of gout, especially to prevent acute gout attacks (Hainer et al., 2014). The initial dose is usually 100 mg/day and can be increased incrementally to a maximum of 900 mg/day to achieve the target blood uric acid level of 6 mg/dl (Perhimpunan Reumatologi Indonesia, 2018). With the population aging, a change toward an unhealthy lifestyle and diet, and increasing access to medicine, the burden of gout arthritis has been increasing (Russell et al., 2020), and therefore, the availability of safe and effective therapy is warranted.

However, allopurinol is recognized as one of the main causes of SCARs, including SJS and TEN. These conditions are associated with high fatality and significant burden on the health system in terms of direct costs of treatment (Gonçalo, 2018). Early detection of risk factors of allopurinol-induced SCARs is very important to establish preventive measures and reduce the number of severe cases (Jung et al., 2015).

Since it was first reported in 2005 (Hung et al., 2005), a growing number of studies conducted in other countries indicate that the HLA-B*58:01 allele is significantly associated with increased risk of developing SJS/TEN in patients using allopurinol (Stamp et al., 2016). In the cases presented, testing for HLA-B*58:01 was positive, which confirmed allopurinol as the causative drug of SJS/TEN. Our cases report confirmed findings from studies that link HLA-B*58:01 and allopurinol-induced SJS/TEN conducted in other Asian countries, including Thailand and Vietnam (Sukasem et al., 2016; Van Nguyen et al., 2017). Our cases involved patients with Javanese ethnicity, for which evidence on the genetic predisposition of allopurinol-induced SJS/TEN has not been established. However, a previous study shows that the polymorphism of HLA genes in Javanese ethnics shared similarities with Southeast Asian populations (Yuliwulandari et al., 2009). The allele frequency of HLA-B*58:01 in Javanese ethnics is around 6%, which is comparable to other Southeast Asian populations (Saito et al., 2016). Another study on allergy to nevirapine also found that the presence of HLA-B*58:01 allele is quite frequent among the study participants (Pudjiati et al., 2016).

Our study supports findings from other studies that HLA-B*58:01 can potentially be used as a pharmacogenetic marker for allopurinol-induced SCARs (Sukasem et al., 2016; Van Nguyen et al., 2017). Thus, the incidence of SCARs induced by allopurinol and other drugs can be prevented if such genetic information is known before any treatment is initiated (Duong et al., 2017). Until now, however, few countries in Southeast Asia have implemented genetic screening prior to initiation of allopurinol treatment despite the high frequency of HLA-B*58:01 among population in the region (Puangpetch et al., 2015; Van Nguyen et al., 2017). A larger study to establish strong association between HLA-B*58:01 and SCARs and provide evidence for policymaking on genetic screening test in Indonesia is warranted.

## Data Availability

The datasets generated for this study can be found in the Indonesian data repository: https://rinarxiv.lipi.go.id/lipi/preprint/view/631.
